# Quality of Life Improvement with Sublingual Immunotherapy: A Prospective Study of Efficacy

**DOI:** 10.1155/2012/253879

**Published:** 2012-01-30

**Authors:** Mary S. Morris, Amanda Lowery, Demetrios S. Theodoropoulos, R. Daniel Duquette, David L. Morris

**Affiliations:** ^1^Allergy Associates of La Crosse and Mayo Clinic Health System Franciscan Healthcare-La Crosse-Onalaska, WI 54650, USA; ^2^Allergychoices Inc., Onalaska, WI 54650, USA; ^3^College of Science and Health, University of Wisconsin-La Crosse, La Crosse, WI 54601, USA; ^4^Allergy and Asthma Center, Hagerstown, MD 21740, USA

## Abstract

Due to its excellent safety profile, ease of administration, and economic considerations, sublingual immunotherapy (SLIT) is becoming a preferred form of allergen specific immunotherapy. The efficacy of SLIT is still debated. The purpose of this act of practice trial is to evaluate quality of life outcomes in patients treated with SLIT. Fifty one patients with allergic rhinoconjunctivitis demonstrated by skin testing completed the Rhinoconjunctivitis Quality of Life Questionnaire (RQLQ) at initiation, at four months and at 10–12 months of SLIT. Significant improvement (*P* < 0.05) on six of seven domain categories of the RQLQ questionnaire was noted. Total RQLQ scores also showed significant improvement. This study supports SLIT as a modality effective in controlling allergic symptoms.

## 1. Introduction

Allergen-specific immunotherapy in the treatment of IgE-mediated allergy has been used for longer than a century; yet, its major form, subcutaneous immunotherapy (SCIT) has not become a widely accepted routine treatment for allergy. Patients will often suffer from severe symptoms and allergic comorbidities before consulting with an allergist or considering immunotherapy. Children especially are unlikely to adhere to SCIT. Subcutaneous injections for immunotherapy are believed to be tedious and unlikely to lead to sustained improvement. Standardization, safety, and efficacy concerns, along with the inconvenience of injections and frequent office visits, keep the vast majority of allergic patients from receiving SCIT. Recruitment to immunotherapy is poor: less than 5% of all allergic patients receive immunotherapy. Compliance is even poorer: among adult patients who agree to undergo SCIT, adherence is disappointing with more than two thirds dropping out within a year of initiation. One tenth of SCIT candidates fail to show up for their first injection [1]. In some countries, the scope of SCIT has been curtailed substantially by administrative decisions [2]. At the same time, a wealth of evidence in literature and clinical practice supports the safety, efficacy, feasibility, compliance, and economic profile of sublingual immunotherapy (SLIT) [3–7]. In the United States, however, SLIT remains uncommon and is only offered by few practices with special interest in this method. With this study, we sought to evaluate the subjective symptom responses of patients treated with multiantigen SLIT. The information provided with the present study may lead to better appreciation of the potential of SLIT and may foster the design of large-scale, multicenter studies for its full appraisal.

## 2. Methods

### 2.1. Subject Selection and Testing

**Figure 1:**
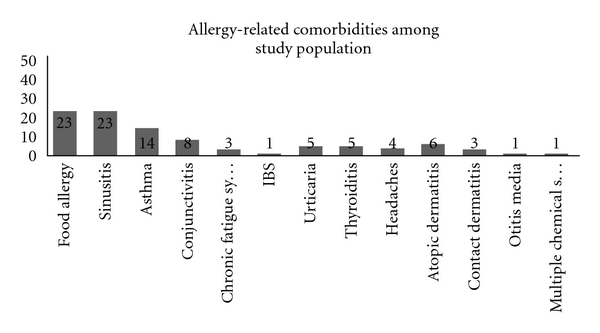
Of the 51 study participants, most had one or more comorbid allergic conditions with the average number of 1.9 chronic conditions per participant.

Subjects were recruited from patients of Allergy Associates of La Crosse, a single specialty practice that has been offering SLIT for 41 years. The study was approved by the Mayo Clinic Health System Franciscan Healthcare-La Crosse, Institutional Review Board. All subjects were diagnosed with allergic rhinoconjunctivitis on the basis of their history and positive skin test results. Skin test positivity was assessed by obtaining a response greater than the negative control and greater than two thirds of the histamine control using intradermal dilution testing (IDT) [8]. Antigens selected for testing were determined by a self-administered patient history questionnaire and initial consultation with their physician. Patients with dermographism or systemic mastocytosis were not included.  Figure 1 also shows that the patient population was affected by one or more comorbid allergic condition upon arrival for their first appointment. Skin test panels included 15–30 antigens representing dust mite, weed, tree, grass, and fungal allergens typical of the northern Midwest (see Table 1). The number of allergen extracts varied by patient, as the number of offending allergens ranged from six to 24 with the mean of 15.15. Round one patient enrollment occurred from July through December and round three visits occurred from January through November, thus crossing multiple peak pollen seasons and limiting the influence of allergen season bias.

### 2.2. Sublingual Immunotherapy Administration

Sublingual immunotherapy based on skin test reactivity was initiated according to the La Crosse Method Practice Protocol [9].

A capital aspect of SLIT, at least as practiced in the United States, is the adjustment of the treating dose to skin reactivity. For this purpose, allergen extracts are serially diluted by decrements of ×5. The purpose of such dilution is to adjust dose to skin reactivity under the premise that adverse reactions (including local reactions) define a level of tolerance. For many patients, skin test reactivity does not necessarily reflect the degree of sensitization. A negative skin test, however, and minimal/absent late-phase responses do establish a de facto threshold of tolerance.

**Figure 2:**
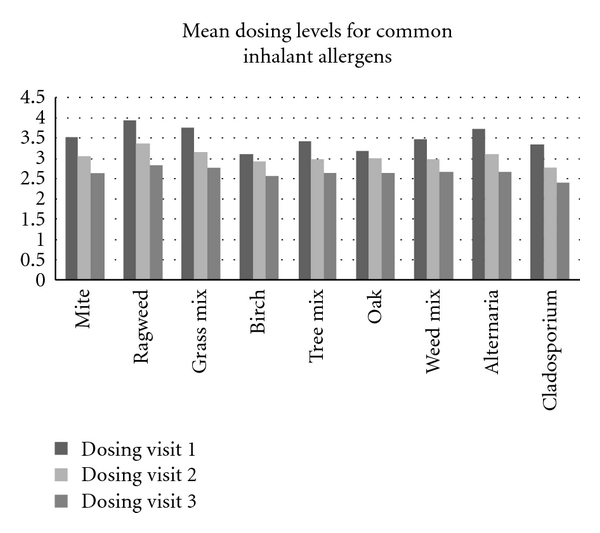
Dosing levels with the La Crosse Method are escalated over the course of treatment based on skin test reactivity. Dosing levels are carefully adjusted in an effort to balance therapeutic benefit without creating unnecessary patient side effects.

Dosing for each patient was tied to skin test results for each individual antigen and adjusted over the course of treatment (see Figure 2). With ongoing treatment, the need to regularly adjust the degree of testing (and dosing) to the long-term effects of immunotherapy is dictated by the fact that, over time, skin test reactivity tends to decline with immunotherapy. Thus, initiation of SLIT at a strength corresponding to the highest dilution that produced a near-negative skin test establishes a safe threshold of tolerance; thereafter, upward titration of immunotherapy doses against declining skin reactivity is used for safe build-up and unnecessary local or systemic reactions.

A skin test of greater than 7 mm using dilution number 7 correlates with the highest level of reactivity. Thus, the lowest dose administered of the offending allergen is dilution number 7. As skin test reactivity improves, doses are escalated to the next allergen dilution until the patient has reached dilution number 1 for his/her different allergens. The starting dose of each individual antigen is titrated based on skin test (or in vitro specific IgE testing) level of reactivity, (see Tables 2 and 3). Sublingual immunotherapy with multiantigen treatment addresses multiple allergies that are specific to each individual patient. Each bottle consisted of a 90-day supply that was individually prepared for the patient using Greer Laboratory and ALK-Abello extracts and compounded in the Allergy Associates of La Crosse clinical laboratory.

Over a 1000-fold range of antigen dilutions have been reported to produce clinical improvement with SLIT suggesting that a straight dose-effect does not exist [10]. Other variables such as dosing frequency, extract quality, and length of treatment also need to be considered. Numerous studies have observed and suggested a limited capacity of the sublingual mucosa and have shown clinical improvement with lower, but more frequent doses [11–13]. Patients were advised to take their sublingual immunotherapy drops three times daily. Given that SLIT is retained in the sublingua for up to 48 hours, administering two to three doses per day is reasonably expected to secure unbroken allergen-exposure and overlap generously with antigen uptake by the dendritic cells and migration to lymphoid organs.

### 2.3. Questionnaire Administration

Symptom severity was evaluated by the Rhinoconjunctivitis Quality of Life Questionnaire (RQLQ). This disease-specific validated questionnaire was developed by Professor Elizabeth Juniper and has been used extensively throughout the world in a large number of clinical trials [14]. New clinic patients were asked to complete the RQLQ at their first visit before the onset of sublingual immunotherapy treatment and two subsequent follow-up visits at three- to six-month intervals. The full-version RQLQ encompasses 28 questions in seven domains (activity limitations, sleep problems, non-nose/eye symptoms, practical problems, nose symptoms, eye symptoms, and emotional function). Patients were asked to recall their experiences during the previous seven day period and to give their responses on a 0- to 6-point scale (none of the time to all of the time). A total RQLQ score is also calculated by adding the scores of the individual domains together.

 This study was a prospective analysis that compiled and compared collected RQLQ data from patients undergoing sublingual immunotherapy. Collected data for each patient were compared to that particular patient's baseline data and two subsequent patient visits for changes in each RQLQ parameter as well as total RQLQ score. Timing of follow-up for visit two ranged from 1.23 months to 10.94 months, with a mean follow-up time of 4.1 months. Follow-up for visit three ranged from 2.82 months to 17.94 months with a mean follow-up time of 7.06 months. The average duration of treatment during the study was 11.19 months.

### 2.4. Statistical Analysis

Descriptive and bivariate statistics were performed using the Standard SPSS data package. Statistical significance was designated as *P* < 0.05.

## 3. Results

### 3.1. Patient Characteristics

Paired RQLQ data were available for 51 patients who were skin tested and started on SLIT. Participants were comprised of 13 males and 38 females, with ages ranging from 22 to 63, and a mean age at initiation of SLIT of 45.8 years.

### 3.2. Quality of Life Results

**Figure 3:**
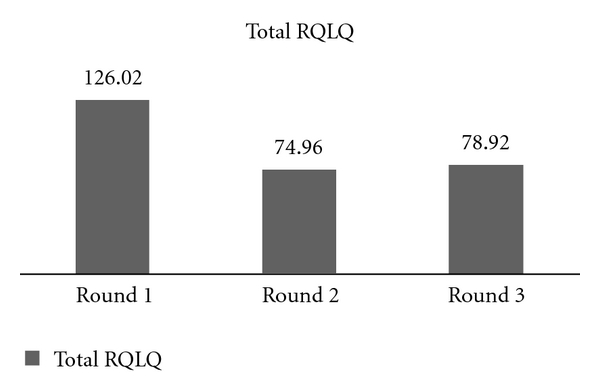
Participants' aggregate RQLQ scores were compared from their initial appointment and follow-up visits one and two. Statistical significance was achieved within four months of beginning sublingual immunotherapy and continued through their second return visit (Round 3). *P* < 0.05. Standard error for Round 1 = 7.74, Round 2 = 6.35, and Round 3 = 7.61.

Paired RQLQ results revealed statistically significant (*P* < 0.05) improvement in six of seven domains evaluated by the RQLQ after four months of treatment. Improvements were seen in the activities, nonnose/eye symptoms, practical problems, nasal symptoms, eye symptoms, and emotional categories. Results are presented in Table 4. Statistically significant improvements were noted in 23 of the 28 overall questions. Furthermore, the total RQLQ for the whole cohort declined significantly (*P* < 0.5) from 126.02 to 74.96 within the first four months of treatment (see Figure 3). 

**Figure 4:**
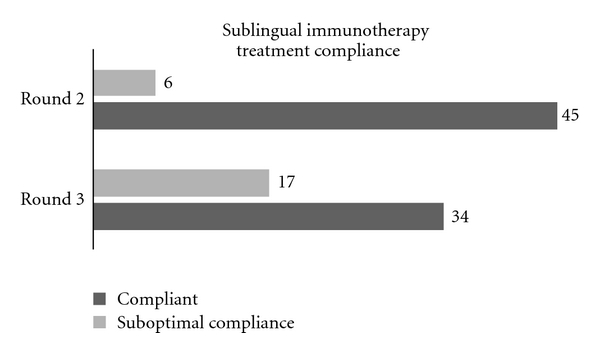
Patients were advised by their providers to take sublingual immunotherapy drops three times daily. Patient records showed a greater adherence to three times daily dosing from their initial appointment to second visit than from their second appointment to third.

 Although just shy of achieving statistical significance, participants that adhered to three times daily dosing of sublingual immunotherapy showed better improvement in RQLQ scores than participants who were suboptimally compliant. Advised compliance to treatment declined from round two to three, which may have affected round two to round three overall RQLQ scores (see Figure 4). Further studies with larger study populations are needed to validate treatment compliance and multiple versus single daily dosing.

## 4. Discussion

A number of studies have demonstrated statistically significant effects on allergic rhinoconjunctivitis and asthma symptoms in SLIT [15–20]. These studies, however, were all heterogeneous and of small magnitude. More importantly since no two studies used the same protocol, the value of their meta-analysis is questionable. They all used single-allergen monotherapy to evaluate efficacy, an approach which does not reflect the sensitization status of patients with allergic rhinoconjunctivitis, and may in fact have led to undertreatment and subsequent under-appreciation of the efficacy of SLIT. The number of SLIT (or even SCIT) efficacy studies employing multiple allergens in the treatment regimen is so surprisingly small that their low number and insufficient data on efficacy have been addressed unfavorably [21]. These studies were characterized by their sporadic nature. They were not followed by subsequent studies that would have established a continuity of approach which might have made up, to some extent, for methodological defects. Significantly, the ultimate end-point, which is improvement of symptoms, was not assessed by a validated instrument, developed by an independent party, such as the RQLQ [9]. To our knowledge, a modified, shortened version of the RQLQ, the mini-RQLQ, has been used in one study employing SLIT for multiple allergens but this study relied on retrospective selection of subjects and only enrolled fifteen patients, thus raising significant questions as to both its power and freedom of bias [22]. Our study is the first prospective study of SLIT efficacy, employing multiple allergen extracts for treatment, a protocol for SLIT which has been applied for 41 years, a validated questionnaire, and a number of subjects large enough to satisfy power requirements.

 In the present study, SLIT, as formulated by the La Crosse Method Protocol, is effective in reducing symptoms and improving quality of life after four months of treatment (see Table 4). This improvement was most prominent in activity, non-nose/eye symptoms, nasal symptoms, and emotional domains. Improvement in the sleep domain of the RQLQ was also observed, but did not reach statistical significance. This improvement was sustained and demonstrated again at 10–12 months of treatment. Given the high compliance rates with the La Crosse Method SLIT, it is expected that the improvement achieved is likely to be sustained and possibly expanded with ongoing treatment beyond the first year. Sneezing and irritability, two parameters, which in a previous SLIT efficacy study employing the mini-RQLQ were found unaffected, are demonstrated to decline in the course of the first four months of SLIT [22].

 The mechanism underlying SLIT has been reviewed [7]. Although not fully delineated, it appears that a systemic alteration of the Th1/Th2 balance is effected in SLIT by the promotion of tolerogenic T-cell clones. Interaction of dendritic cells with naïve T-cells is necessary for this change to occur. Production of TGF-*β*, IL-10, and possibly other regulatory cytokines appears to be critical. Ongoing changes may in some cases be reflected in skin reactivity as well as in specific IgG and IgE production changes. The protocol used in the present study may be well suited to effect these changes. It can be summarized in three cardinal points: (i) initial and thereafter regular titration of treating SLIT doses against skin reactivity and symptom response with skin reactivity meant as a biphasic response whose late phase reactions are also taken into account; (ii) frequent administration of SLIT doses to secure continuous, maximal, and uninterrupted saturation of the sublingual dendritic cells' potential for phagocytosis and migration, that is, three doses per 24 hours; and (iii) maintenance of allergens in high glycerin solutions in order to prevent decay and suppress proteolytic activity [9].

 In summary, this study represented a preliminary attempt to investigate the effectiveness of multiantigen SLIT in a complex patient base. Experience with this protocol over the years has been rewarding and has shown clinical benefit with a wide variety of allergic conditions including advanced respiratory disease in adults with mold allergy [23], asthma prevention in pediatric patients [24], and contact allergies including nickel [25] and poison ivy [26] while maintaining a remarkable paucity of adverse reactions of any significance. The present study underscores the efficacy of SLIT; however, we recognize that the absence of a placebo group limits the interpretation of results. Given the large number of patients currently treated and high rates of compliance, multicenter, controlled studies are needed of greater magnitude and expanded scope to include morbidities and associations such as recurrent/chronic sinusitis, atopic dermatitis, gastroesophageal reflux, and migraines. Sustained suppression of symptoms after eventual completion of SLIT will also need to be studied.

## 5. Conclusion

Statistically significant reduction of symptoms and improvement of quality of life are demonstrated during the initial four month period of SLIT. After the first four months, reduction of symptom scores is sustained and continuous. These data support the efficacy of SLIT and need to be followed by controlled trials to evaluate the efficacy of this method.

## Figures and Tables

**Table 1:** Sensitivities detected by skin testing. Grass mix includes Kentucky Blue/June, Meadow Fescue, Orchard, Perennial Rye, Redtop, Sweet Vernal, and Timothy. Tree mix includes American Beech, American/Eastern Sycamore, American Elm, Black Walnut, Black Willow, Eastern Cottonwood, Red Oak, Red/River Birch, Shagbark Hickory, Sugar/Hard Maple, and White Ash. Weed mix includes Cocklebur, Lamb's Quarter, Common Mugwort, Pigweed (rough/red), and Dock/Sorrel Mix (red/sheep and yellow dock).

Allergy Associates common environmental allergens and the percentage of participants testing positive to the following.	
Dust mites	51 (100%)
Ragweed	51 (100%)
Grass mix	48 (94%)
Birch	42 (82%)
Tree mix	50 (98%)
Oak	37 (73%)
Weed mix	42 (82%)
Alternaria	50 (98%)
Cladosporium	49 (96%)

**Table 2:** Serial dilutions are then used to expand La Crosse Method doses from one to seven.

Antigen	La Crosse method concentrate	Concentration number 1 dilution
Pollens	1 mL 1 : 20 w/v	1 : 100 w/v
Mold	1 : 20 w/v	1 : 100 w/v
Mite mix	1 mL conc + 2 mL diluents for 10,000 AU/mL	2000 AU/mL
Cat	1 mL + 4 mL diluents for 2000 BAU/mL	400 BAU/mL
Epithelias (except cat)	1 : 20 w/v	1 : 100 w/v
Grass mix	100,000 BAU/mL	20,000 BAU/mL
Bermuda grass	10,000 BAU/mL	4000 AU/mL
Short ragweed	100,000 AU/mL	20,000 AU/mL

**Table 3:** How 1 : 5 serial dilutions are made: from La Crosse Method number 1 dilution.

1 mL of dilution number 1 + 4 mL of diluent = dilution number 2	
1 mL of dilution number 2 + 4 mL of diluent = dilution number 3	
1 mL of dilution number 3 + 4 mL of diluent = dilution number 4	
1 mL of dilution number 4 + 4 mL of diluent = dilution number 5	
1 mL of dilution number 5 + 4 mL of diluent = dilution number 6	
1 mL of dilution number 6 + 4 mL of diluent = dilution number 7	

**Table 4:** Mean RQLQ Scores Presublingual Immunotherapy and at Subsequent Visits.

Category sum	Round 1		Round 2		Round 3	
	Mean (SE)		Mean (SE)		Mean (SE)	
Activity sum	8.14	(.57)	4.92*	(.44)	4.63*	(.52)
Sleep sum	5.84	(.68)	3.61	(.45)	3.92	(.63)
Non-nose/eye sum	17	(1.37)	10.59*	(1.04)	10.71*	(1.22)
Practice problem sum	8.43	(.65)	4.63*	(.50)	5.06*	(.54)
Nasal symptom sum	11.69	(.74)	7.25*	(.60)	7.63*	(.64)
Eye symptom sum	8.92	(.82)	5.49*	(.67)	6.16*	(.67)
Emotional sum	10.55	(.87)	4.27*	(.59)	4.82*	(.67)

*Denotes RQLQ domains found to have a statistically significant decrease in symptom scores throughout the duration of sublingual immunotherapy treatment. *P < 0.05.
